# Facilitators and barriers to adherence to urate-lowering therapy in African-Americans with gout: a qualitative study

**DOI:** 10.1186/ar4524

**Published:** 2014-03-29

**Authors:** Jasvinder A Singh

**Affiliations:** 1 Medicine Service, Birmingham VA Medical Center, 805B, 510 20th Street S, Birmingham, AL, USA; 2 Department of Medicine at School of Medicine, and Division of Epidemiology at School of Public Health, University of Alabama, Birmingham, AL, USA; 3 Department of Orthopedic Surgery, Mayo Clinic College of Medicine, Rochester, MN, USA

## Abstract

**Introduction:**

Limited literature exists for qualitative studies of medication adherence in gout, especially in African-Americans. The aim of this study was to examine the facilitators and barriers to adherence to urate-lowering therapy (ULT) in African-Americans with gout.

**Methods:**

In this study, nine nominal groups lasting 1 to 1.5 hours each were conducted in African-Americans with gout, six with low ULT and three with high ULT adherence (medication possession ratios of <0.80 or ≥0.80, respectively). Patients presented, discussed, combined and rank ordered their concerns. A qualitative analysis was performed.

**Results:**

This study included 43 patients with mean age 63.9 years (standard deviation, 9.9), 67% men, who participated in nine nominal groups (seven in men, two in women): African-American men (n = 30); African-American women (n = 13). The main facilitators to ULT adherence (three groups) were the recognition of the need to take ULT regularly to prevent gout flares, prevent pain from becoming chronic/severe and to have less dietary restriction; the lack of side effects from ULT; trust in physicians; and avoiding the need to seek emergent/urgent care for flares. Patients achieved high ULT adherence by organizing their pills using the pillbox and the incorporation of ULT intake into their routine to prevent forgetting. The main barriers to optimal ULT adherence were (six groups): doubts about effectiveness of ULT, concerns about cost and side effects, concomitant medications, forgetfulness, refilling the prescriptions on time, pill size and difficulty in swallowing, competing priorities, patient preference for alternative medicines (that is, cherry juice) and frequent travel.

**Conclusions:**

Identification of facilitators and barriers to high ULT adherence in African-Americans with gout in this study lays the foundation for designing interventions to improve ULT adherence in racial minorities.

## Introduction

Gout is a chronic, inflammatory arthritis that affects 3.9% of U.S. adults [1]. Principles of appropriate long-term gout management include the appropriate use of urate-lowering therapy (ULT) to keep serum urate (sUA) at or below 6 mg/dl and to treat acute gout flares with short-term anti-inflammatory drugs such as corticosteroids, colchicine or non-steroidal anti-inflammatory drugs (NSAIDs). ULT options include xanthine oxidase inhibitors, such as allopurinol (used by the majority) and febuxostat, with a much lower use of uricosurics, including probenecid [2-4]. Appropriate use of ULT is central to the achievement of target sUA < 6 mg/dl. Target sUA achievement is associated with a lower risk of gout flares, tophi and medical care costs [5-10] and is recommended by gout guidelines [11,12]. A key gap in gout management is the lack of appropriate use of ULT, characterized by a suboptimal adherence to ULT and the use of lower than needed doses of ULT [9,13]. Of all rheumatic conditions leading to quality of life deficits, gout is one of the most well understood biochemically and pathophysiology and perhaps most amenable to good control and remission; yet it is successfully treated infrequently. This is at least partially due to our lack of understanding of why patients fail to use effective, cheap treatments.

Recent quantitative research that used surveys showed numerous patient knowledge gaps with regards to the use of ULT and other medications for gout [14]. Two recent qualitative studies of semi-structured interviews with 26 US Caucasian patients with gout and 20 UK Caucasian gout patients (15 men, 5 women) found that gout patients reported non-adherence with allopurinol and cited several reasons, such as financial concerns, forgetfulness and doubts about the benefits of long-term treatment [15,16]. Both studies were done in Caucasian men, covered a broad array of topics ranging from the gout treatment to quality of life to patient knowledge and so on with relatively little focus on medication adherence, which is a key challenge in gout management [9,13]. Two large knowledge gaps exist in qualitative studies in medication adherence in gout, since none of the previous studies: (1) recruited gout patients with high medication adherence or stratified patients by medication adherence (for example, medication possession ratio); and (2) recruited racial minorities.

African-Americans share a disproportionate burden of gout compared to Caucasians, since the: (1) prevalence of gout is higher (5% vs. 4%) [1]; (2) likelihood of treatment with ULT including allopurinol is lower [17,18]; (3) non-adherence with ULT is higher (odds ratio, 1.86) [19]; and (4) baseline serum urate (7.9 vs. 7.1) is higher and odds of achieving target serum urate levels <6 mg/dl are lower (odds ratio, 0.67) [18]. To our knowledge, there are no studies investigating the reasons for poor medication adherence in African-Americans with gout. None of the previous studies recruited patients with high medication adherence to understand the facilitators of high ULT adherence.

Why do patients not take the gout medications they are prescribed? Medication adherence depends greatly on how patients perceive their disease/illness and what meaning they attach to the disease and the treatment [20,21]. Meanings are important and fundamental in their interpretation of disease and treatments [22]. Corbin and Strauss proposed a model for chronic disease consisting of three components, namely Body, Biographical time and Conceptions of self, that is, the BBC chain [23,24]. The model indicates that only when these three components are in balance, interactively stabilizing and reinforcing one another, do people feel healthy and well [24-26]. Body failure and interference with identity-relevant functions by chronic diseases can lead to destabilization of the BBC chain, leading to a patient having the sense of being ill. Chronic diseases can interfere with plans, performances and meanings, which formerly contributed to valued appraisals of self, resulting in loss of previously valued self-image, described as “loss of self” and, subsequently, social isolation [23]. Patients with chronic diseases are often motivated to take medications to treat the disease to avoid or reverse illness-induced loss of self and to restore their disrupted biographies. On the other hand, medication itself may lead to body failure, be perceived to either cause or be associated with body failure or interfere with identity-relevant function (for example, dizziness as a medication side effect, preventing a patient from social activities), in which case patients will have low medication adherence or will stop the medication.

Our objective was to better understand the impact of medication and illness meanings as well as biographical disruption due to disease on the medication use and adherence in gout. To our knowledge, there are no qualitative or quantitative studies focused on medication adherence in African-Americans with gout who have a disproportionate burden of the disease and higher rates of non-adherence [1,17-19]. Our study aim was to assess the facilitators and barriers to adherence to ULT in African-Americans with gout, based on this chronic disease model.

## Methods

### Patients

Patients were eligible for the study if they had had one or more outpatient visit with an International Classification of Diseases, ninth revision, common modification (ICD-9-CM) code for gout, that is, 274.xx, between January 2011 to September 2012 at the Kirklin Clinic, a community-based outpatient clinic. Patients were screened on the telephone for the presence of gout, whether they were taking or had taken allopurinol and/or febuxostat (the most common urate-lowering therapies (ULTs)) for the treatment of gout, and regarding self-reported adherence to ULT (<80% vs. ≥80%, corresponding to low vs. high adherers). The University of Alabama at Birmingham’s Institutional Review Board (IRB) approved the study. Patients provided verbal consent (the need for written consent was waived by the IRB) and received free parking, light refreshments and a $30 check for study participation.

### Nominal group sessions

A nominal group technique (NGT) is a variant of the traditional focus group method that aims to identify the overall opinion of a group. The NGT is a structured process aimed at developing an inclusive list of issues related to a specific question, then soliciting feedback on the relative importance of these lists through rank-ordering procedures [27-29]. NGT capitalizes on the participants’ experiences and skills. The NGT has proven successful in soliciting useful information from experts, professional caregivers and patient groups for a variety of conditions [27,30-35]. An advantage of NGT is that it promotes more even participation rates and the input from all group members is equally weighted. Therefore, the data generated from NGT usually provide a more valid representation of the implicit views of the group than would be achieved with a traditional focus group format. Thus, while the focus groups are helpful in understanding the breadth of an issue, the NGT is an excellent tool to address a specific key question in-depth. It is recommended that five to nine participants constitute each nominal group. A question was posed to the group after receiving extensive feedback on the wording of the question from gout patients in the clinic and clinicians - What keeps you from taking your allopurinol or Uloric (also called febuxostat) everyday?

All nine nominal groups were held at the outpatient clinics at the Kirklin Clinic, in patient education rooms that are set up with a white board, a round table with chairs and an area for refreshments. The PI and the nominal group leader (JAS) welcomed the participants, and after brief introductions, explained the purpose of the study, and wrote the single key question for the nominal group on a flip chart, which was also used to capture key concerns and patient discussion. Patients were also provided the question on a blank piece of paper. Research associates (BA, AB, AO) provided administrative support and assisted in taking notes and transcripts.

Each nominal group lasted 60 to 70 minutes and the NGT process was divided into four discrete steps. Each member wrote down as many responses as possible, usually in short phrases to the key question in the first 10 to 15 minutes, independently and quietly. Next, the nominal group leader asked each member in a round-robin fashion to state an idea from his or her list and wrote it on a flip chart placed before the group, indicating each idea with a unique letter from A to Z. No discussion was permitted until all ideas had been listed. Subsequently, each item was discussed in an interactive group format and group participants consolidated items when applicable. Next, all participants were given five index cards and were asked to identify and rank-order the five responses deemed most important from 1 to 5, 5 being the highest score. The participants indicated their preference for important items by rank ordering. The outcome of the process was the mathematical aggregation of each member’s score with the highest score corresponding to the top ranking theme for the group.

Rank-ordered results from each nominal group were mapped and compared. Similar to the within-group process, responses were analyzed based on the number of groups identifying responses with high relative rank ordering. The transcriptions were examined to identify all statements made relative to each response and created a comprehensive list of statements (as in the tables). These are presented in the tables and results section.

## Results

Nine nominal groups were conducted with African-Americans, three with higher adherers and six with low adherers to ULT. Overall, there were 30 African-American men (seven nominal groups) and 13 African-American women (two nominal groups) and mean age (SD) was 63.9 (8.4). Several themes emerged from the nominal groups, in high adherers (three groups) vs. low adherers (six groups) that are summarized in the two sections below.

### Facilitators to ULT adherence in African-American gout patients with high adherence

We conducted three nominal groups with 14 African-American gout patients (all men) who had >80% MPR for ULT. Mean age was 66.3 years (SD, 8.4), and 100% were men. The main reasons why they took ULT regularly were as follows (also, see Table 1). The first two themes were consistent with perception of a positive meaning of the medication and a positive effect of optimal treatment on body and identity-relevant functions (mapping to the BBC model).

**Table 1:** Facilitators for medication adherence to allopurinol of febuxostat among African-Americans with gout

	**Quotes**	**# Votes**
**Group 1 –African American men (four patients)**		
Adversity to pain	“If I don’t take it everyday, it (gout) would be back in my foot and anywhere.”	20
	“It makes the pain stay away.”	
	“Gout is super painful- I’ll do whatever avoid that pain.”	
	“It hurts so bad, when I had gout.”	
	“I have been shot- I’d rather be shot again than have the pain due to gout.”	
It is a habit	“It’s simple, once a day.”	9
	“Once a week, I put all my medications in a compartment in the pill box.”	
	“It’s simple-take it once in the morning.”	
	“Put the pill in the napkin and take it to work.”	
	“It’s only 1 out of the 10 medications and it’s easy.”	
It solved my problem	“When it first happened, I thought I hurt myself, then I was diagnosed with gout; allopurinol solved the problem.”	8
Avoid surgery/complications from other medications	“I had surgery from gout once and it is helping me avoid another- they drained gout from my foot.”	7
	“Helps avoid prednisone- put on 60 pounds in 30 days.”	
I do not recognize any bad side effects	“I don’t see any side effects.”	7
Can go places that I want to go	“Free to go anywhere”	5
	“Can go to movie, park”	
	“Have my mobility”	
	“Makes me stay out of bed”	
Allopurinol gives me a less restrictive diet	“Before allopurinol, certain foods triggered gout. Now I can eat anything I want in moderation.”	4
**Group 2: African-American men (six patients)**		
I take allopurinol to stop the pain	“Prevent, minimize flare-up.”	26
	“To be pain-free.”	
	“Help keep the pain under control.”	
	“I hurt so bad, I have to take it.”	
	“The pain is hell.”	
	“The pain was so bad, I couldn’t even walk.”	
	“That’s a given.”	
It is a habit	“I have a habit.”	14
	“(it’s a) routine”	
	“I take my meds in the morning- I wake up dry and take my meds with water in the morning.”	
	“I was taking my meds irregular, then over time it became regular.”	
	“Being consistent with my blood pressure med made me consistent with my allopurinol.”	
Allopurinol is a good medicine that my family practice doctor can prescribe	“I don’t have to see a specialist.”	13
	“It costs more and I have to drive longer to see a specialist, if I have to go to specialist; (I go) when a medication other than allopurinol is needed.”	
When I am fine, my family is also	“Helps to keep the family to do things that they want to do.”	8
	“If I am ok, my family is ok, everything “clicks”.”	
To keep my mobility	“…To keep me walking.”	8
	“Gout hit me so bad, both sides, I could do nothing; now I can do things.”	
	“Helps me walk inside and outside.”	
	“Activities I’d like to do.”	
	“I am very active, helps me stay active.”	
	“Let’s me do exercise, bicycling.”	
	“I can go on the treadmill.”	
	“Affects my quality of life positively.”	
I purchase my meds (including allopurinol) every 90-days, it helps me not run out	“If I get 30-day prescription, I keep running out.”	7
	“I do mail order for 90-days.”	
	“I get it at Costco.”	
	“90-day is cheaper than 30-day.”	
I use a pill organizer, and organize my pills every two weeks	“ I have been using it (pill organizer) for years, that’s the one thing that helped, “that nailed it.”	7
	“Just do it.”	
	“Keep it on the track.”	
	“Very effective.”	
	“You go back and see if you took it- sometimes I find out I didn’t take it.”	
	“It becomes more or less a protocol.”	
Allopurinol is an everyday medication	“This is a medication, we have to take every day to keep it in our system.”	6
	“If you get off for 2–3 days, it (gout) is coming back.”	
Allopurinol gave them a less restrictive diet	“I can eat most things I want.”	3
Group 3: African-American men (five patients)*		
I was requested by my doctor	“I have faith in my doctor.”	17
	“Think doctors are here for a purpose.”	
	“My doctor knows the best.”	
	“Trust.”	
Take allopurinol to stop the gout from hurting	“The thought of flare-up (is scary).”	17
	“The thought of the condition getting worsening, the pain becoming more severe…”	
	“Keeps me from having to rush to doctor’s office.”	
	“You have to take it.”	
	“Since being on allopurinol, I haven’t had a flare for 2 years.”	
	“Take allopurinol to prevent the handicap.”	
	“The thought of condition spreading to other foot.”	
Because it helps to keep my uric acid in check	“The reason gout come is that uric acid builds up in the bone; Now uric acid is where it is supposed to be, that’s why my gout is away.”	14
I do not want to report to my doctor that I was not taking my allopurinol	“.. expectations from my doctor (My doc has set expectations).”	8

*4 patients voted, one had to leave prior to voting.

1. ULT prevented gout flares and pain: All three groups listed this among their top facilitators. They indicated that they took their ULT regularly to be pain-free, keep gout under control, minimize flare-ups, prevent them from rushing to the doctor’s office for acute flares, prevent the pain from becoming chronic or severe and prevent handicap due to gout. One patient, who served in the armed forces, said “I have been shot- I’d rather be shot again than have the pain due to gout”*.*

2. ULT gave them a less restrictive diet: Two of the three groups indicated this among the top facilitators. Patients said that ULT intake allowed them to eat the foods they like in moderation without having the flare-ups.

3. Habit/routine: Two of the three groups indicated this among the top facilitators. They mentioned that taking their ULT was part of the routine, that they took their ULT along with other medications in the morning or the evening, use a pill box to organize their ULT and that a simple once a day intake makes it easier to take this medication.

Other reasons cited by one nominal group each included the following: ULT helped keep the chronic pain from gout under control and to avoid the condition from getting worse and pain from becoming more severe; ULT avoided complications from other medications such as prednisone; ULT was not associated with any bad side effects; patients trusted their health care provider’s recommendation; ULT was easily prescribed by their primary without the need to go to a specialist; and that it helped the patients keep their uric acid under check (Table 1).

### Barriers to ULT adherence in in African-American gout patients with low adherence

In six nominal groups of 29 African-American gout patients with a mean age (SD) of 62.5 (7.6) years (range, 60 to 75 years; 16 men and 13 women) with suboptimal adherence to ULT, we explored the reasons for poor adherence to ULT, that is, barriers to ULT adherence. The following were the top themes, identified as barriers to ULT adherence, among the six nominal groups (also, see Table 2). The first four themes were consistent with the patient’s perception of the meaning of the medication and its association with body failure or interference with identity relevant functions.

1. Not convinced that the ULT medication is effective: All six groups listed this among their top concerns. Patients reported that they were doing great without ULT medication, if gout had subsided they did not need to take the ULT medication, other medications, such as indomethacin, worked better for their gout, gout flare-ups continued despite taking ULT, they did not know whether or not to take their ULT medication during an acute gout flare, they were not sure whether medication was effective in preventing gout flares, they skipped the medication when they felt better with their gout, they were not sure whether the medication worked for their gout, they decided whether or not to take the ULT based on how they felt, and they did not feel a difference in their symptoms when they missed ULT for a few days.

2. Side effects: Five of the six groups listed this among their top concerns. Patients reported mainly gastrointestinal side effects such as gastritis, abdominal pain, bad taste in the mouth, belching, nausea, vomiting and diarrhea, and drowsiness.

3. Concomitant medications and intake of too many pills: We combined these two inter-related concepts and four of the six groups listed this among their top concerns. Patients were concerns about taking too many pills and associated frustration, taking too many milligrams in total being detrimental to their body, side effects from other medications (nausea) making it difficult for them to take the ULT and medication interactions.

4. Pill size and swallowing difficulty: Two of the six groups listed this among their top concerns. Patients complained about the large size of the pill, the need to break the pill to swallow it, problem swallowing due to vocal cord disease and the need to swallow one pill at a time when we are prescribed multiple pills per day due to the dose needed.

5. Cost: Five of the six groups listed this among their top concerns. Patients were concerned about copayment with limited resources and the need to fill multiple medication prescriptions, the expensive nature of the ULT (especially febuxostat), the need to ration the pills due to limited income and the challenges with copayment after retirement or losing job insurance which previously had covered the ULT.

6. Refill issues: Four of the six groups listed this among their top concerns. Patients reported they often ran out of prescriptions, could not easily figure out which one was due for refill due to their taking multiple medications, threw away the bottle before calling it in, did not order the refill in time, had difficulty picking up the prescription from the pharmacy, or felt lazy regarding getting the refill in time.

7. Forgetting to take the ULT medication: All six groups listed this among their top concerns. Patients reported forgetting because of other things they had to do, travel related to their work, being on vacation, the fact that they take so many other pills, trouble reading labels due to vision problems and the interruption of their daily routine.

**Table 2:** Barriers to allopurinol or febuxostat adherence among African-Americans with gout

	**Quotes**	**# Votes**
**Group 4: African-American women (six patients)**		
Not convinced that the medication is effective and should be taken daily	“I felt it wasn’t helping me.”	20
	“When I missed a few days nothing changed.”	
	“Taking it did not make any difference.”	
	“If it is doing good then why should you have to take additional medications?”	
	“I would miss days and not feel any different from when I was taking it.”	
	“Maybe it was a dosing issue?”	
	“I have intermittent flare ups and I have learned to use colcrys as a treatment.”	
Forgetting to take the medicine	“I’m not going to forget the gout pill during an active attack. It more of a mental thing (to forget the routine otherwise) that I think its not doing any good.”	21
	“Sometimes I will just plain forget because I am busy etc.”	
	“If I forget I will take it later in the day. I go ahead and take it because I do not want to flare up.”	
	“I will forget because I am taking so many other medications.”	
	“Can’t remember if I have taken it that day so I will not take an extra because I do not want to take double.”	
	“If your daily routine is interrupted then it is easy to forget because you are used to taking it at the same time each day.”	
	“Did I take it, did I forget?”	
Side effects	“I have gastritis. Switched my times taking it now and night with milk and I have not had anymore episodes.”	15
	“Sometimes it will make me sick. I will throw up.”	
	“I just got tired of being sick on my stomach.”	
	“You may wonder what you have eaten but it only happens when you take that pill.”	
	“I had abdominal pain.”	
	“…(it) caused diarrhea.”	
Other concomitant medications	“Too many pills to take for other conditions.”	14
	“Additional gout medications and still having flares.”	
	“All of my medications are grouped together. If I miss one I miss them all.”	
	“I keep thinking about the milligrams that I am taking. I don’t want to hurt my body. I am taking to many milligrams.”	
	“Sometimes I feel “lazy” because I take so many pills.”	
Concern about drug interactions	I don’t know what affect the other medicines have on my gout medications. Is that what’s making me sick. I don’t know.	11
Cost	“I have to be able to get my other prescriptions, and this gives me trouble due to cost.”	5
	“Trying to balance between my medications and my daughters meds makes it very difficult.”	
Travel and planning for travel	“Sometimes I am out of town, and did not have my medication with me.”	4
	“I take my whole bottle so that I will not do without.”	
	“My medication come from CVS so I can get them from most places in the US.”	
**Group 5: African-American women (seven patients)**		
Cost	“My insurance does not cover this.”	27
	“I am on fixed income, $77 for 1 month, sometimes, I don’t have money for copay.”	
	“Doctor has to call in the medication despite the prescription- due to insurance company.”	
Forgetting to take the medicine	“Problem with eyes, reading the label is not easy taking too may other medications.”	21
	“Usually took it every day before work; Sunday and Saturday is an issue, since I don’t go to work.”	
	“Out of routine.”	
Other concomitant medications	“I get nausea from other medications.”	13
	“Gout pill is the last one I take. So when I am nauseous from others, I skip.”	
Too many medications	“I did not want to take it because I was taking so many other medications.”	10
	“I am tired of taking medications.”	
	“Recently have had very high BP in 200 s, got a lot of pills that required more allopurinol.”	
Medication refill issues	“It may have been that I could not pick up the prescription from the pharmacy.”	9
	“When I ran out, did not fill it as soon as I could.”	
	“Felt lazy.”	
	“Too may things to do…”	
	“I have negligence…”	
Alternative medicine choices	“I read cherry juice was god, so I took cherry juice instead of allopurinol.”	3
	“I don’t like to take medicine.”	
	“I tried eating cherries…”	
Not convinced that the medication is effective and should be taken daily	“Not hurting in weeks, So I thought I did not need to take it.”	6
	“If I skip it for a week, I feel flare coming on, but less than that can’t see a difference.”	
Large pill size and vocal cord problems with swallowing	“Pills are big, now taking 5 other pills.”	16
	“I break the pill up due to swallowing problem.”	
	“Sometimes have problem swallowing because of vocal cord problems.”	
**Group 6: African-American men (three patients)**		
Side effects	“It makes me sluggish along with my other medications- not knowing which medication causes drowsiness.”	11
	“Bad taste in mouth.”	
	“Belching”	
	“I am a retired entertainer, you don’t want to be drowsy, you want to be alert.”	
	“I used to play at church. You can’t do that when you are sluggish. I say “Amen, I am out of here.”	
Too many medications	“I have so much on my mind due to the all the pills I have to take.”	9
	“My mind comes and goes, I was in the military.”	
	“Getting frustrated with the routine of taking so much medication- I just want to stop.”	
Not convinced that the medication is effective and should be taken daily	“When I feel better, I don’t take it.”	8
	“If everything is good, you feel better in the morning – I don’t need it today.”	
	“If I could not do it today (take my allopurinol), I’d be ok.”	
	“If I feel better, I think I don’t need it today.”	
	“If better in the morning, I take my allopurinol in the afternoon.”	
Forgetting to take the medicine and confusion	“When I don’t see in the pill box, I miss it.”	8
	“Usually I set pills out at night; I take 12 pills, so I have 2 pill boxes, sometimes I forget which day it is and forget to take it.”	
	“I have a pill box too, if I am feeling ok, I am not going to take 2–3 of them.”	
	“Sometime I can’t remember, because I took so many pills.”	
	“I take 6 pills a day- sometime I can’t remember, because I take so many pills.”	
	“I take 7 pills, if I am tired, I can’t remember, whether I took it.”	
Cost/economic impact	“Very expensive.”	5
	“Copay was $20- I have to ration the pills; I go with the pill I need the most, when I do that.”	
	“I pay $40 co-pay.”	
	“I live in the retirement home; so (what I have left for) my rent goes up or down with my copays.”	
	“My income changed all of a sudden, when I lost my job, it was difficult.”	
Pill size	“Pill too big”	3
	“Sometimes it’s hard for me to swallow, I take 600 mg.”	
	“The 300 mg pill is bigger than 100 mg. I had to take a lot of water to wash it down.”	
	“Have to take one pill at a time.”	
Side effects and problems with taking it on an empty stomach	“When the pain is very severe, I can’t eat and I don’t take pills without eating.”	1
	“Makes your stomach queasy.”	
	“I took allopurinol later in the day without food, stomach starting feeling bad.”	
**Group 7: African-American men (five patients)**		
Forgetting to take the medicine	“It could be business travel, just a busy day, or hey I forgot that day. I have started putting my medicine in the weekly pill distributor. I also take blood pressure medicine and will forget it from time to time. I will forget my gout medication more regularly. Both must be ordered and for some reason I will remember to order the blood pressure medication more often. I don’t tend to forget it.”	16
	“It is easy to forget on a vacation day.”	
Medication causing the flares	“(allopurinol) caused me to have big flare where I had to go to the emergency room.”	14
	“I just don’t take it. - the attacks were coming too frequently. To the point I could not get out of the bed.”	
Not convinced that the medication is effective	“Would work at times but at others it would not.”	11
	“I would still have flare ups. (I had been on it for at least 2 months then again within the first 30 days).”	
Side effects	“…Made me nauseated. – felt like I wanted to throw up.”	10
	“Hard on the stomach.”	
	“It just don’t feel right.”	
	“Its better when I drink milk, but I’m not sure if that is causing me to flare more.”	
	“Continuously in the bathroom issue. (Diarrhea) after the second day in my system it would start.”	
	“Diarrhea at least once a day.”	
Refill issues	“….mainly my fault.”	10
	“I just didn’t order it in time.”	
Cost/economic impact	“Uloric worked fine with the gout flares, but due to my insurance the medication was expensive. Additional paperwork is required yearly to get it approved.”	8
Too many pills	“Plus taking it on top of all the other mediation I take (high blood pressure etc.).”	6
	“Too many meds in my system.”	
	“Started off twice a day moved to once.”	
	“With the other medications it just does not do me any good.”	
**Group 8: African-American men (six patients)**		
Not convinced that the medication is effective and should be taken daily	“It don’t work for me.”	27
	“Took Indocin for years that helped. I took allopurinol, didn’t help, I was having more pain while taking allopurinol compared to when I was taking Indocin. Indocin worked better.”	
	“It took too long to clear up the pain.”	
	“I would still have flare ups. I had been on it for at least 2 months.”	
	“If gout has subsided, I did not need to take it, so I stopped.”	
Side effects	“Because of the side effects of allopurinol.”	17
	“Because of the gout infection in my elbow: My elbow got sore, I was told to take allopurinol, pain got worse, got fluid out of the elbow, diagnosed with infection, told it was due to allopurinol, got antibiotics in the hospital and then at home –have not taken allopurinol since then.”	
	“The doctor took me off of it due to side effects.”	
Cost/Economic impact	“Money- It was Money.”	12
	“Have to take a lot of meds, copay went up, have to choose.”	
Refill issues	“I forget to refill.”	11
	“I threw away my bottle before I calling.”	
	“Sometimes forget to go and pick up from pharmacy.”	
	“I have so many meds that I take, its’ not easy (to remember which one is due for refill).”	
Forgetting to take the medicine	“Forget to take it.”	8
	“Forget to take it on time.”	
	“Sometimes on the go, can’t take any of my medications.”	
	“Most of the time, I take my medication after breakfast, if I don’t see the pill box at breakfast, I forget at times.”	
**Group 9: African-American men (two patients)**		
Not convinced that the medication is effective	“Doing great without medication.”	9
Refill issues	“Prescription ran out- didn’t fill it.”	9
Competing priorities	“Working all the time.”	5
	“I have to get to my jobs and getting to work (keeps me busy).”	
Forgetting to take the medicine	“Keep forgetting (to take it).”	5
	“…because of other things I have to do.”	

One nominal group each brought up the following in their top seven concerns, with the first two being consistent with identity-relevant functions.

1. Issues related to travel and the need to plan ahead of time: Patients reported not having their ULT with them when they were out of town. They also indicated that they needed to plan ahead to take their medication with them or access it through a retail pharmacy in another town/place when they were traveling.

2. Medication causing flares: Some patients reported big flares when they started taking ULT that made them go to the emergency room or experiencing attacks too often after starting ULT.

3. Patient preference for alternative medicine: Patients indicated that they decided to take cherry juice instead of allopurinol, or eating cherries.

4. Competing priorities: Patients cited being busy with multiple jobs and the need to work all the time as barriers to the regular intake of ULT.

## Discussion

To our knowledge, this is the first qualitative study to assess facilitators and barriers to ULT adherence in African-Americans with gout and the first study to enroll gout patients with low and high ULT adherence. The main barriers to optimal ULT adherence were doubts about the effectiveness of ULT, concerns about cost and side effects (short-term and long-term cumulative), the impact of concomitant medications and of taking too many pills, forgetfulness, challenges with refilling the prescriptions on time, pill size and difficulty in swallowing, competing priorities, patient preference for alternative medicines (such as cherry juice or extract) and frequent work-related travel. The main facilitators to ULT adherence included patient realization that they needed to take ULT regularly to prevent gout flares, to prevent the severe pain and handicaps associated with acute gout and to prevent pain from becoming chronic/severe, the patients organizing their pills and their routine so that they would not forget to take their ULT, the patient’s ability to have fewer dietary restrictions and the ability to eat foods they liked in moderation once they were taking ULT regularly, lack of side effects, avoidance of side effects from alternatives such as corticosteroids, trust in physicians, and avoiding the need to seek frequent emergent/urgent care or a subspecialist’s care. Several findings from this study deserve further discussion.

It is now well-known that compared to Caucasians, African-Americans not only have a higher prevalence of gout [1], but also have more severe gout with higher baseline sUA, worse ULT adherence and lower likelihood of ULT treatment and the achievement of target sUA <6 mg/dl [17-19]. This disproportionate burden of gout and the lack of any previously published studies on racial minorities were the key motivations for our study.

Several findings in this study are novel. Both low and high adherers to ULT identified issues related to body medication, identity-relevant functions (interference or facilitation) and meaning imparted to the mediation as key facilitators and barriers to ULT adherence, that fit our theoretical model, that is, the trajectory model with the BBC chain [23,24]. The themes were opposite in several common domains. A key difference in low vs. high adherers was their understanding of what the medication could/would do for them. The low adherers were not convinced that ULT was needed every day or was helping their gout vs. high adherers who had the very opposite experience and perception, that is, the ULT helped them avoid severe pain and suffering. Low adherers cited lack of knowledge and communication from physicians regarding the usefulness of ULT, an important observation from our study, complimenting a survey study finding that knowledge deficits were common in gout patients [14]. High adherers had greater trust in their physicians and saw the adherence to ULT as a way to avoid more doctor visits, keep their uric acid levels in check and prevent chronic joint damage that reflected more knowledge about gout and treatments.

The current study also provides an in-depth examination of ULT adherence in African-Americans. Some quotes from patients were eye-openers; for example, one veteran who had served in the armed forces said, “I have been shot. I’d rather be shot than have my gout attack again”, signifying the severe pain of gout and its impact on patients and why it is so important to adequately treat gout. This study adds knowledge to the area by identifying additional previously not-described barriers, such as the impact of concomitant medications and of taking too many pills, ULT pill size, swallowing difficulty, patient preference for alternative medicines, travel-related and refill issues, and competing priorities. These findings indicate that non-medication alternatives are considered true alternatives to pharmacotherapy by patients and should be discussed with patients with gout. Now that these barriers have been identified, a further study into how to develop interventions targeting these barriers is needed. Patient education and patient-physician communication can target several barriers and has the potential to improve the outcomes in African-Americans with gout. Gaps in patient and physician knowledge have been demonstrated in addition to their perception of treatment related issues in gout, including non-adherence [15]. This indicates that education initiatives may also need to target both patients and health care providers to reduce the differences in perceptions.

Other key findings from this study based on studying facilitators to high ULT adherence, may help educate low-adherers and design interventions. African-American gout patients, who were our success stories (high-adherers) differed from those with poor ULT adherence (low-adherers) in several key aspects, which also map well to the Health Belief Model [36]. This model emphasizes that individuals can change their behavior if they consider themselves susceptible to disease (chronic pain), realize the disease’s consequences (frequent flares) and perceive that engagement in a particular behavior (ULT adherence) will be beneficial in preventing negative disease consequences and disease severity (preventing flares, pain, suffering [36]. Compared to low adherers, gout patients with high ULT adherence: (1) understood their disease severity, including gout related pain and suffering, better (better perception of disease severity and personal susceptibility); (2) did not have misconceptions regarding ULT and had a better perception that ULT benefits outweigh risks (side effects), that is, considered the balance of perceived barriers to treatment and benefits from treatment favorable to them and (3) had effective cues to prompt action of regular ULT intake (using a pillbox; taking medications at the same time; health belief model). Thus, they had overcome the same barriers that were faced by their peers that had poor adherence to ULT. Thus, if an intervention can incorporate ways to encourage several behaviors practiced by the high-adherers, it has the potential to improve ULT adherence and gout outcomes.

Our study is the first to confirm that several barriers to optimal allopurinol use in Caucasians [15,16] are also barriers to optimal allopurinol use in African-Americans. In a qualitative study of 26 Caucasian patients with gout, during the phone interviews, gout patients cited financial concerns, forgetfulness, worsening of gout, potential side effects, lack of information and doubts about the length of treatment, as reasons [15]. In another study of 20 UK gout patients (15 men, 5 women), during the semi-structured interviews with gout patients that covered a wide array of topics, patients reported non-adherence often due to concern about the side-effects or the belief that gout did not warrant any long-term treatment [16]. Our study design did not allow us to compare the barriers to gout medication adherence by gender. This is an important gap in our knowledge that we plan to investigate next. Other studies have shown gender differences in medication adherence with women having slightly lower adherence than men [37-39]. It remains to be seen if this is true in the case of gout as well, a predominantly male disease.

Our study has several limitations. While we reached saturation for our low-adherers with the six nominal groups, one may wonder if we reached saturation in high-adherers. We were able to identify and successfully recruit fewer patients with ULT adherence of 80% or more in our study, even after extensive telephone screening. However, several themes were noted to be repeating in the current sample of high adherers, supporting the notion that saturation can be attained with two to three nominal groups, as it seemed to be in our case. The number of high adherers recruited in this study is the largest nominal group sample studied to date. Second, this in-depth study has findings applicable to African-Americans with gout. We oversampled for women with gout (another understudied population) to improve the study’s generalizability. Generalizability to Caucasians was not our goal, since at least two small studies exist in Caucasians and our focus was disadvantaged minorities. However, several themes from earlier studies with Caucasians were replicated in this study, indicating some applicability to Caucasians. Study strengths included study of a difficult to reach, underserved and understudied group that bears a disproportionate burden of gout and the use of NGT to obtain an in-depth answer to the specific study question.

## Conclusion

In conclusion, this study provides an in-depth insight into facilitators and barriers to ULT adherence in African-Americans with gout. Several new themes were identified in this study. This new knowledge should serve as a foundation for research of behavioral and non-behavioral interventions to improve ULT adherence in minorities with gout. Such studies have the potential to improve gout care and outcomes in patients suffering from this disease.

## Abbreviations

BBC chain: Body, Biographical time and Conceptions of self; ICD-9-CM: International Classification of Diseases, ninth revision, common modification; IRB: Institutional Review Board; NGT: Nominal group technique; NSAIDs: Non-steroidal anti-inflammatory drugs; SD: Standard deviation; sUA: serum urate; ULT: Urate-lowering therapy

## Competing interests

There are no financial conflicts related directly to this study. JAS has received research and travel grants from Takeda and Savient, and consultant fees from Savient, Takeda, Regeneron and Allergan.
